# Novel Wearable Optical Sensors for Vital Health Monitoring Systems—A Review

**DOI:** 10.3390/bios13020181

**Published:** 2023-01-23

**Authors:** Baljinder Kaur, Santosh Kumar, Brajesh Kumar Kaushik

**Affiliations:** 1 Department of Electronics and Communication Engineering, Indian Institute of Technology Roorkee, Roorkee 247667, India; 2 Shandong Key Laboratory of Optical Communication Science and Technology, School of Physics Science and Information Technology, Liaocheng University, Liaocheng 252059, China

**Keywords:** wearable sensors, optical sensors, biofluids, optical wearable sensors, bio-receptor elements

## Abstract

Wearable sensors are pioneering devices to monitor health issues that allow the constant monitoring of physical and biological parameters. The immunity towards electromagnetic interference, miniaturization, detection of nano-volumes, integration with fiber, high sensitivity, low cost, usable in harsh environments and corrosion-resistant have made optical wearable sensor an emerging sensing technology in the recent year. This review presents the progress made in the development of novel wearable optical sensors for vital health monitoring systems. The details of different substrates, sensing platforms, and biofluids used for the detection of target molecules are discussed in detail. Wearable technologies could increase the quality of health monitoring systems at a nominal cost and enable continuous and early disease diagnosis. Various optical sensing principles, including surface-enhanced Raman scattering, colorimetric, fluorescence, plasmonic, photoplethysmography, and interferometric-based sensors, are discussed in detail for health monitoring applications. The performance of optical wearable sensors utilizing two-dimensional materials is also discussed. Future challenges associated with the development of optical wearable sensors for point-of-care applications and clinical diagnosis have been thoroughly discussed.

## 1. Introduction

Human-wearable sensors [1] are among the most cutting-edge technologies that have been investigated for a variety of applications, including health monitoring, remote monitoring, home rehabilitation, ailment detection, on-site monitoring of pesticide residues, and medical treatment efficacy [2,3,4,5]. Wearable sensors that monitor blood pressure, oxygen level, temperature, glucose level, body fluids, pulse and heart rate, electrolytes, drugs, hormones, and metabolites are currently commercially available in the form of smart watches, gloves, patches, tattoos, facemasks, wrist bands, clothing, and glucometers [6,7,8,9]. Sensors are classified into different categories depending upon biofluids, targets, bio-receptor elements (BRPE), and different sensing platforms. Biofluids consist of sweat, tears, saliva, interstitial fluid, metabolites, electrolytes, hormones, and proteins. BRPE are nucleic acids (NA), proteins, and synthetically designed polymer receptors [8,10]. In comparison to other biofluids, tears, saliva, interstitial fluid (ISF), and sweat are the most extensively studied due to their accessibility from body parts in a non-invasive manner and their low risk of infection and damage. For various sensing applications, sensing platforms consist of optical, electrochemical (ECL), surface-enhanced Raman scattering (SERS), and mass-based sensor designs [1,11,12]. Wearable sensor platforms include both physical and chemical sensors. The former measures and monitors pressure, humidity, shear, strain, and temperature, while the latter measures chemical concentrations in various fluids, such as biomarkers, pollutants, and toxins [9,13,14,15]. Various optical technologies that include fluorescence, colorimetric, photoplethysmography, and SERS are being explored for wearable on-site detection of various bio-chemical samples [16]. Umapathi et al. summarized the various on-site colorimetric sensors to detect the pesticides present in agricultural foods [5]. Wearable sensors have various applications in aerospace that includes eye-tracking glasses, radio headphones, oxygen masks, and helmets for airplane manufacturing applications, cardiac monitoring device, human cognitive states in-flight monitoring, air quality management [17]. They have also been explored to detect various substances that include hydrogen, hydrocarbons, gases such as oxygen, nitrogen, nitrogen oxides, carbon monoxide, and carbon dioxide, and various engine emissions have been developed [18].

**Figure 1:**
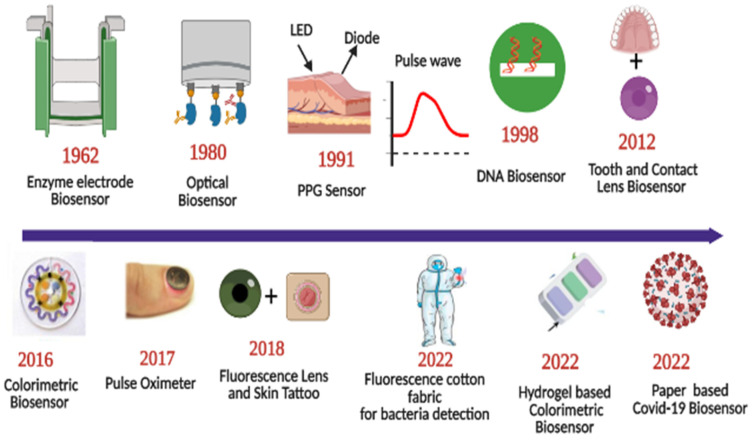
Roadmap of progress of wearable optical sensors. Reprinted with permission from *Science translational medicine*, Copyright 2016, American Association for the Advancement of Science [26]—Reprinted with permission from Advanced functional materials, Copyright 2017, Wiley [27]—Reprinted with permission from Advanced Healthcare Materials, Copyright 2022, Wiley [28]—Reprinted with permission from ACS Applied Nano Materials, Copyright 2012, American Chemical Society [29].

**Figure 2:**
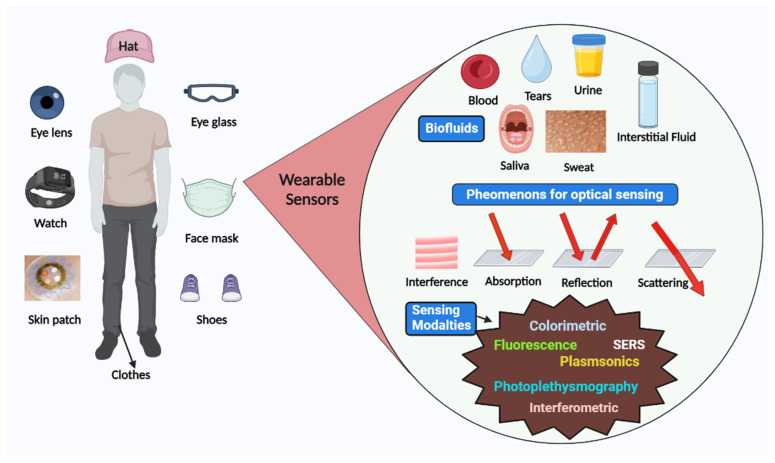
Schematic representation of various available wearable optical sensors, sensing mechanism, and biofluids for detection.

Sensors have a substrate that serves as the foundation for other components, signal conversion devices, and the analyte of interest. BRPEs are utilized to enhance the affinity of a device for a particular target [19]. Figure 1 provides a brief summary of the evolution and history of wearable sensing technologies since the advent of the optical sensor in 1980. In recent years, extensive research has been conducted on two-dimensional (2D) materials to increase sensors’ performance through signal amplification [20,21,22,23]. Metal nanoparticles (NPs), nanomaterials derived from carbon, MXenes, core–shell hybrid nanocomposites, and synthetic biomaterials are included in this study [24,25]. Due to the development of several synthesis and fabrication technologies, non-invasive health monitoring has developed [26,27,28,29]. Wearable sensors are difficult to implement in real-world applications due to the minute quantities of various fluids, such as interstitial fluid, tears, and sweat, produced at low rates (1 nL/min/mm^2^) and evaporating rapidly [30]. This issue can be resolved by integrating polymer- or fabric-based microfluidic channels with a sensor design due to their high flexibility, biocompatibility, and stretching ability [31]. Figure 2 depicts the commercially available wearable sensors that include eyewear, a wristwatch, a facemask, a skin patch, clothing, and footwear that utilize various biofluids. In addition, it consists of numerous sensing platforms based on scattering, reflection, absorption, and interference, as well as sensor modalities based on various phenomena.

## 2. Different Sensing Platforms, and Monitoring Parameters

ECL and optical sensors are the most commonly used transducers and are commercialized for a variety of applications, including heart rate and pulse rate monitoring, glucose, and lactate, whereas the commercialization of optical sensors presents challenges. Due to the development of various modes of ECL sensor design, such as potentiometric, conductometric, voltammetric, and amperometric, the detection of multiple analytes in biofluids has attracted considerable interest. The potentiometric sensor consists of ion-selective electrodes and a reference electrode. The potential of the reference electrode is independent of analyte concentration, whereas the potential of ion-selective electrodes varies with analyte concentration. Various potentiometric sensor designs have been reported for the detection of different ions that include Na^+^, K^+^, NH_4_^+^, Mg^2+^, Ca^2+^, sweat, urine, and tears. Conductometric is the least studied ECL sensing modality, and it has not yet been implemented in wearable sensing. Voltammetry and amperometric methods have been extensively studied for a variety of health monitoring and other applications. The sensor’s three-electrode configuration consists of a working electrode, a reference electrode, and a counter electrode. In voltammetric sensing, a time-dependent voltage is applied to the reference electrode for analytes that are reduced or oxidized in order to evaluate the response between the working and reference electrodes. Amperometric sensing has been investigated for health monitoring utilizing saliva and other fluids containing glucose, lactate, uric acid, ion concentrations, and bacteria. An optical sensor can overcome the numerous limitations of an ECL sensor, such as its BRPE-dependent stability, sensitivity, and accuracy, as well as interference caused by the presence of other substances.

An optical sensor measures chemical or biological responses such as changes in light absorbance, fluorescence, scattering, and color change [31]. In colorimetric sensors, a shift in wavelength in the absorption spectra is observed as a result of the binding of the target with the relevant recognition molecule, which alters the chromophore’s electronic state. Fluorophores are utilized in fluorescence sensors to monitor the absorption and emission of light energy. In a colorimetric sensor, color changes are visible to the naked eye, whereas a fluorescence sensor requires a sophisticated light source to analyze the phenomenon. Colorimetric sensors suffer from several challenges, including the unavailability of highly sensitive reactants and reliable dyes integrable with different textiles or wearable assays from a commercial point of view. Fluorescence sensors are more sensitive than colorimetric sensors to detect different analytes [3]. SERS-based wearable sensors are devices with ultrahigh sensitivity, and SERS activity depends on the distribution of hotspots due to the highly localized field and broad range of excitation angles. Due to the numerous deformations caused by body movements, the production of SERS sensors is a challenging task [32,33]. SERS permits multianalyte sensing, detection of a single molecule, and low analyte concentrations for point-of-care applications. The SERS sensor structure is comprised of a substrate used for field enhancement and a probe molecule adsorbing on the surface of the substrate. The SERS enhancement mechanism consists of a charge transfer-derived chemical mechanism, an electromagnetic enhancement mechanism, and their combinations [34,35]. SERS-based sensors have been extensively investigated for chronic muscle diseases, such as Parkinson’s and Alzheimer’s, microorganisms, and biomolecules [36,37,38]. Recently, plasmonic substrates have been investigated for the development of wearable SERS sensors, as these structures boost the Raman signal emitted by the target molecules [39]. An external light source and spectrometer are required for wearable SERS-based sensors to excite Raman scattering in the target sample and collect scattered light [39].

## 3. Components of Wearable Sensors

The key components of a wearable sensor are flexible substrates, sensing structures that convert one type of signal into another type, and target analytes. To improve the performance of a sensor, self-assembled monolayers, porous materials, and hydrogel or polymeric coatings are required. MXenes are another type of 2D material that can form nanoparticles, composites, single or multiple sheets, with flexible, stretchable, highly conductive, and oxidation-resistant properties [40,41]. For various wearable sensor designs, a variety of 2D materials, including carbon and carbon-derived nanomaterials and transition metal dichalcogenides-based materials, have been investigated.

### 3.1. Substrate Materials

Substrate materials support the various sensor structure components and provide flexibility, stability, durability, light weight, and anatomical compatibility with the human body. The sensitivity and selectivity of the sensor are directly dependent on the surface of the sensor’s structure. In recent years, numerous efforts have been made to improve the sensor surface with chemical, biological, and advanced fabrication techniques, such as inkjet printing, roll-to-roll printing, and gravure printing [3]. The development of fabrication technologies is dependent on the substrate’s mechanical and thermal properties, the bending and folding capabilities of active layers, and its ability to absorb moisture. In the design of wearable sensors, polydimethylsiloxane (PDMS), polyethylene terephthalate (PET), polyimide, silicones, hydrogels, chitosan, parylene, cellulose substrates such as paper, textiles, and unconventionally used leather are extensively explored. Common examples of textile substrates include silk, cellulose nanofibers, cotton, wool, and sponge [13,14,15,16,17,18,19,20,21,22,23,24,25,26,27,28,29,30,31,32,33,34,35,36,37,38,39,40,41,42]. Hydrogels are chitosan- and cellulose-based, cross-linked polymers with high biodegradability and porosity. In recent years, self-healing materials, such as supramolecular polymers, have been investigated in the design of wearable biosensors. For fabricating substrate materials, screen printing, stamping, roll-to-roll, gravure printing, inkjet printing, and 3D printing are the most common techniques. Paper is a popular substrate material for wearable sensors employing capillary force, such as microfluidic devices, paper strip assays, and lateral flow assays. Ion gels are being investigated for their use in wearable electronic sensors as a result of their stretchable properties and capacity to immobilize ionic liquids in a polymer matrix. The substrates used for skin should meet requirements such as a wearable power source, a long wear time, less skin irritation, and easy removal without causing skin damage. Polymers such as polydimethylsiloxane, polyethylene, and terephthalate have good stretchability properties with low toxicity, light weight, and excellent hydrophobicity. Polyimide is used as a substrate in wearable sensors due to its high tensile strength and flexibility at high temperatures. Cellulose paper is widely used in medical applications due to its biocompatibility, flexibility, low cost, spongy texture, and recyclable properties. In the case of skin-based wearable sensors, materials such as hydrogels, silicones, acrylics, and hydrocolloids are being explored as adhesives due to their long use time and ease of removal [43]. A highly stretchable and conductive silicone rubber composite comprised of single-walled carbon nanotubes and silicone rubber composites for wearable sensor applications is also being explored [44]. This composite is unaffected by mechanical punching and has a high tensile strain of nearly 200% and a high conductivity of 18 S/cm as a result of nitric acid doping [45]. Single-walled carbon nanotube and silicone rubber composites increased their elasticity by up to 300%, making them a prime candidate for numerous applications [45].

### 3.2. Sensing Unit

The optical wearable sensor measures various changes, including chemical, physical, and biological, by measuring the change in wavelength, phase, and intensity. This section provides a comprehensive summary of the various wearable sensors and strategies reported in the literature.

#### 3.2.1. Colorimetric Sensors

**Figure 3:**
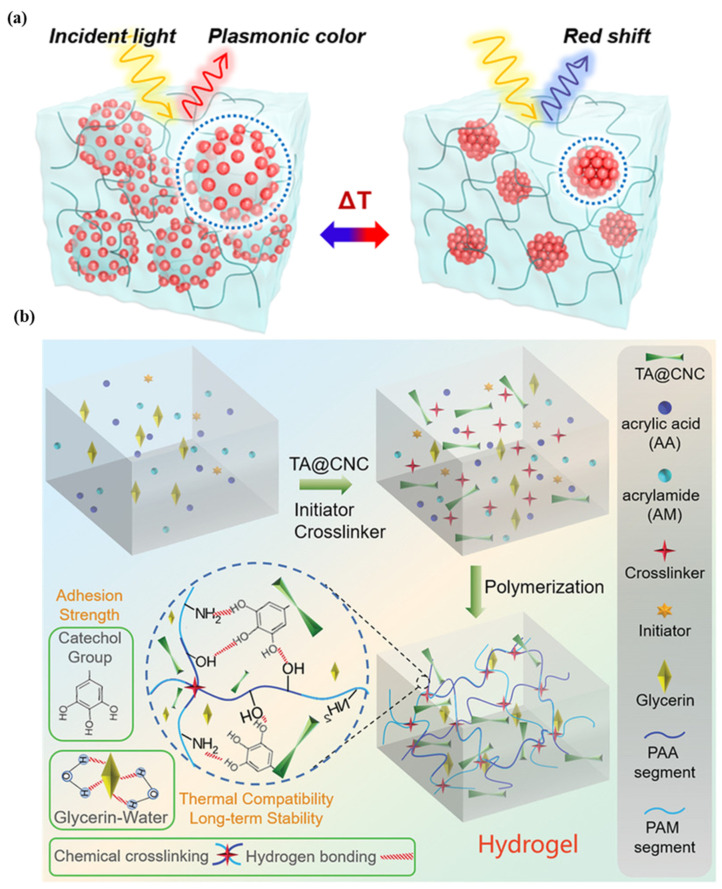
Illustration of (**a**) plasmonic microgels-based patch and patch attached to various parts of the human body. Reprinted with permission from NPG Asia Materials, Copyright 2018, Nature Publishing Group [46]; (**b**) reprinted with permission from Advanced Healthcare Materials, Copyright 2022, Wiley [28].

For the purpose of health monitoring, a hydrogel-based, epidermal, multi-signal sensor is demonstrated. Figure 3a shows the hydrogel-based colorimetric sensor. The hydrogel is produced by coating cellulose nanocrystals with tannic acid, polyacrylic acid, and polyacrylamide in various binary solvents, such as glycerin and water. After 45 days of long-term storage at 20 °C, the material has good adhesion, stretchability, and transparency. This system monitors pH, temperature, and light simultaneously [46]. In the case of colorimetric sensors, there is less contact with the skin, and the sweat rate cannot be quantified. The super-absorbent polymer valves are developed to collect, store, and chemically analyze sweat released by eccrine glands. This platform detects chloride ion concentration via chelation of mercury (II) and ferrous (II) ions [47]. A colorimetric sensor integrated with a microfluidic platform was developed to monitor sweat biomarkers, including chloride, glucose, pH, and lactate concentrations, as well as sweat rate, loss, and temperature (32–37 °C). The measured chloride, glucose, pH, and lactate concentrations are 25–100 mM, 25–100 µm, 5.0–6.5, and 5–20 mM, respectively [48]. A microfluidic paper-based colorimetric design for glucose monitoring was reported based on a saliva sample. Two detection zones, one for glucose and the other for nitrite assays, are interconnected by microfluidic channels to a sampling zone. It provides detection limits of 27 μM for glucose and 7 μM for nitrite. Patients suffering from periodontitis or diabetes were analyzed [49]. A microfluidic channel-based colorimetric sensor capable of measuring sweat glucose and the enzymatic oxidation of o-dianisidine was demonstrated. Sweat is collected from the epidermis layer via a microchannel and transferred to a chamber equipped with a valve, eliminating the possibility of chemical backflow. Glucose oxidase is encapsulated within a microchamber. This sensor has a 0.03 mM detection limit and a linear range of 0.1–0.5 mM for sweat glucose solutions [50]. A colorimetric biosensor comprised of Whatman filter paper and pegylated AuNPs for measuring sweat glucose concentrations in the range of 0.01–0.15 mM. With a low detection limit, 0.01 mM, low glucose concentrations can be detected [51].

A colorimetric as well as fluorescence-based wearable face mask sensor that uses freeze-dried, cell-free reactions for direct nucleic acid detection, i.e., SARS-CoV-2 detection, was developed with the help of nasopharyngeal sampling.

Clustered regularly interspaced short palindromic repeats (CRISPRs) technology is used for specific detection of the analyte. This sensor is light weight ~3 g made up of silicone elastomer, without power source, provide output in two hours at ambient temperatures. In the case of a colorimetric sensor, cellulose substrates and elastomer assembly are used, while for a fluorescence design, the sensor is activated and immobilized in polymeric fiber [52]. A thermo-sensitive, colorimetric skin patch was developed using a plasmonic microgel embedded in a hydrogel film. As depicted in Figure 3b, raspberry-shaped plasmonic microgels are developed by decorating poly (N-isopropylacrylamide) microgels with gold nanoparticles (AuNPs) in order to effectively change the color. The plasmonic microgels patch exhibits a significant extinction peak shift at 176 nm in less than one second with a temperature-sensing resolution of 0.2 °C [28,29,30,31,32,33,34,35,36,37,38,39,40,41,42,43,44,45,46,47,48,49,50,51,52,53].

#### 3.2.2. Fluorescence Sensors

**Figure 4:**
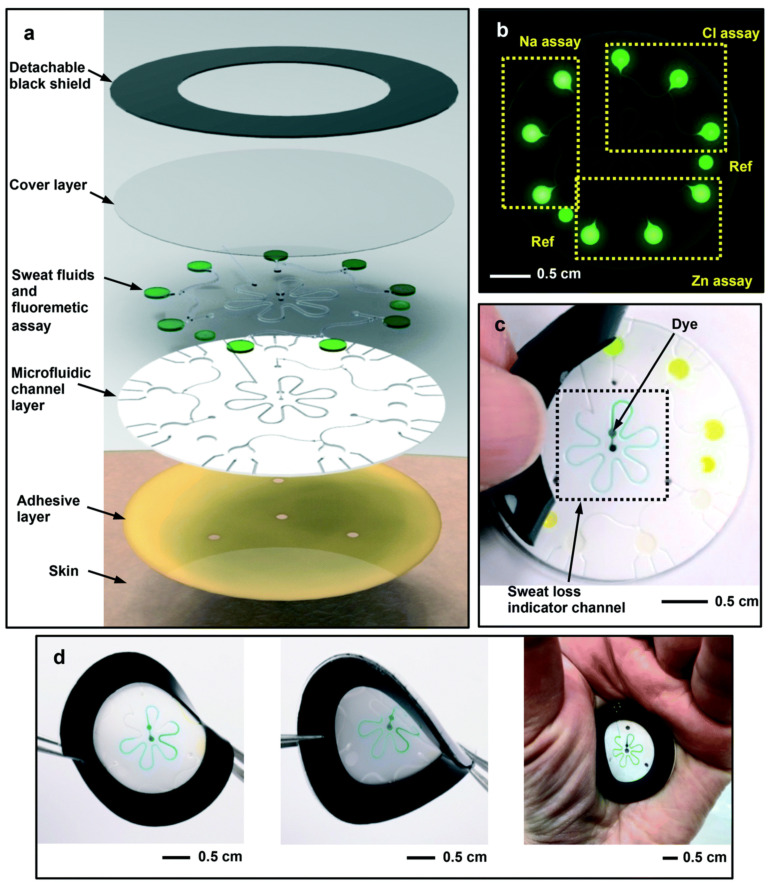
Schematic design of the (**a**) microfluidic fluorescence sensor in to detect the chloride, sodium, and zinc present in the sweat sample; (**b**) fluorescence response of different components; (**c**) peeling of black silicone shield from the sensor surface; (**d**) twisting properties of the device. Reprinted with permission from *Lab on a Chip*, Copyright 2018, Royal Society of Chemistry [54].

**Figure 5:**
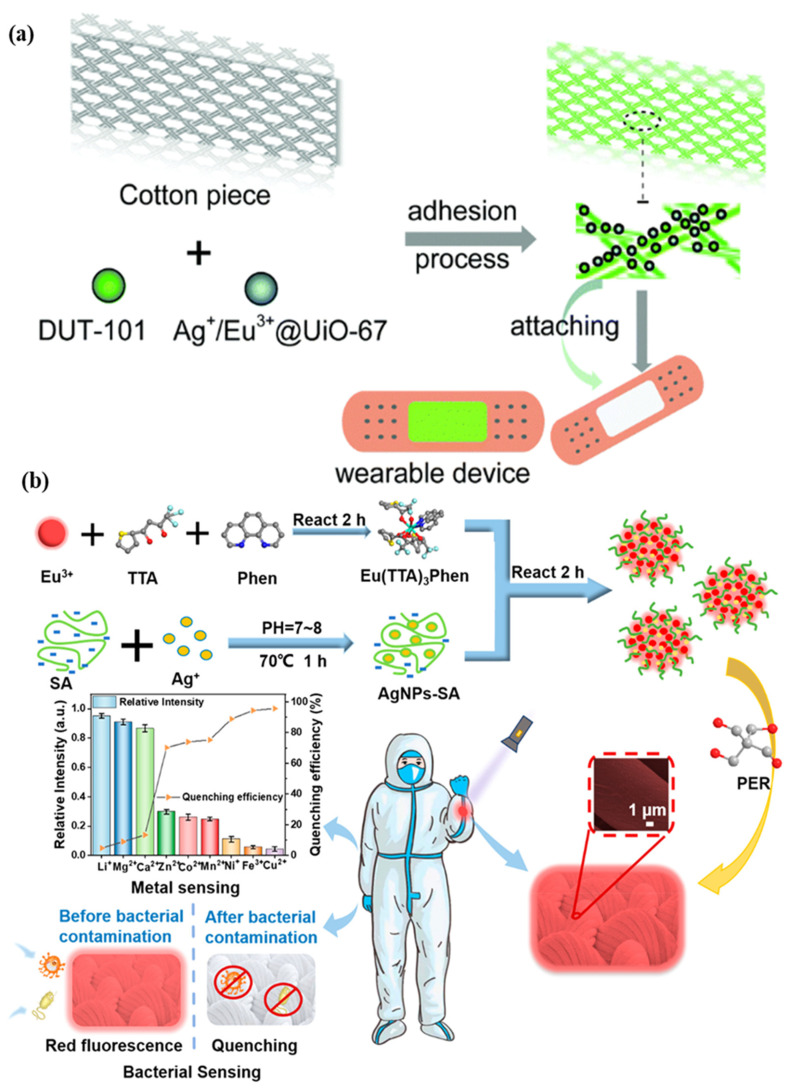
Illustration of (**a**) a metal–organic framework-based wearable sweat monitoring fluorescent device. Reprinted with permission from Journal of Materials Chemistry C, Copyright 2018, Royal Society of Chemistry [55]; (**b**) a fluorescent sensor for detecting Escherichia coli and Staphylococcus aureus pathogenic bacteria. Reprinted with permission from ACS Applied Nano Materials, Copyright 2012, American Chemical Society [29].

A microfluidic biosensor employing a fluorometric sensor was developed to determine the concentrations of sweat chloride, sodium, and zinc, as depicted in Figure 4a. Samples and fluorometric reagents were placed inside the micro-chamber. As perspiration and fluorescence formed a mixture, the light-shielding film was removed from the fluorometric microchambers, and the optical module of a smartphone was utilized to capture fluorescent images of the microchambers. In the optical setup, a smartphone with a dark box was used, which consists of a filter for a specific wavelength range of excitation light. In order to determine the fluorescence intensities of the excited light without interference from the excitation light, another colored emission filter was utilized. The mixture of ionic liquid and fluorometric reagents is mixed using the two different circular microchambers. Image-measured fluorescent intensities of micro-fluorometric chambers were used to determine the concentrations of analyte samples, as shown in Figure 4b. Figure 4c shows a black silicone layer deposited on the surface of the sensor that the fluorescence probes from the photo-bleaching. Figure 4d shows the twisting properties, i.e., forward, backward, and on the palm twisting [54]. A low-cost almost USD 0.03 cellulose-based fluorescence wearable patch was designed for the multi-sensing of glucose, lactate, pH, chloride, and volume that serve as sweat biomarkers. The device is comprised of paper substrates and cotton-based microfluidic channels that collect and transfer sweat samples to paper. The concentration of sweat ranges from 0 to 90 mM, with detection limits of 5 mM, 8 μL, 7 μM, and 0.4 mM for chloride, volume, glucose, and lactate solutions, respectively [55]. Figure 5a demonstrates a fluorescence-based wearable sensor for detecting chloride ions in sweat samples, an indicator of cystic fibrosis. Porous cotton is used as a biocompatible host material with improved absorption capacity, and fluorescent materials such as Ag+/Eu^3+^@UiO-67 and lanthanide metal–organic frameworks improve the color intensity and measurement precision. The fluorescence sensor design demonstrates a high degree of selectivity and sensitivity, as well as a detection limit of 0.1 mM in 35 s [56,57]. As shown in Figure 5b, Eu^3+^-induced sodium alginate/silver (Ag) nanoparticle aggregate is affixed to a fluorescence cotton fabric-based fluorescent sensor for the detection of Gram-negative *Escherichia coli* and Gram-positive *Staphylococcus aureus* pathogenic bacteria. AgNPs with a high specific surface area and a broad spectrum of bactericidal effects are assembled into europium-induced sodium alginate nanoparticles. The complexes of europium emit a strong and distinct red color light emission in the visible spectrum via 4f–4f electron transition while excited using ultraviolet light. This sodium alginate solution gives AgNPs excellent biocompatibility and uniform size [29]. An optical contact lenses-based fluorescent sensor was developed to monitor the glucose solution in tears for the concentration range of 23 μM–1.0 mM through a smartphone. The sensor structure consists of a reference probe grafted into the 2-hydroxyethyl methacrylate hydrogel network of the contact lenses. The fluorescence of images can be controlled using a mobile phone, and the glucose concentration can be monitored by capturing images. This sensor provides an extremely sensitive detection limit of 9.3 μM [58].

#### 3.2.3. Plasmonic Sensors

**Figure 6:**
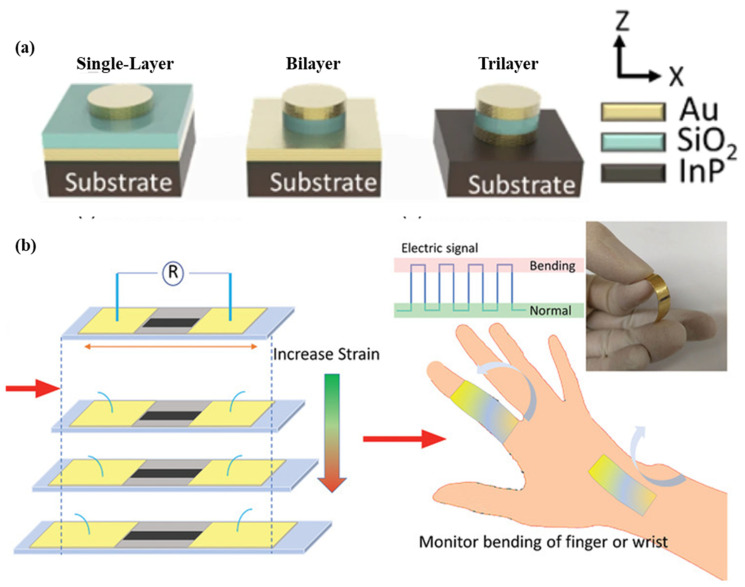
Schematic of the (**a**) flexible MIM-disk-based LSPR cancer sensor. Reprinted with permission from Scientific reports, Copyright 2018, Nature Publishing Group [59]; (**b**) graphene oxide and AuNPs based wearable LSPR strain sensor, Reprinted with permission from Advanced Materials Technologies, Copyright 2021, Wiley [60].

As shown in Figure 6a, a nano-disk-based metal-insulator-metal sensor for detecting A549 cancer cells is demonstrated. The sensor is produced using the localized surface plasmon resonance phenomenon. Polydimethylsiloxane is used as a substrate material, and nano-disks of different geometries are used to enhance the performance of the sensor. The sensor structure contains a 50 nm-thick Au disk, a 60-nm-thick SiO_2_ disk, a 50 nm-thick Au disk, and a 240 nm-thick SiNx adhesion layer. Single, bilayer, and trilayer-MIM-disk LSPR sensors were developed for cancer cell detection. Out of the three structures, the trilayer-based MIM disk structure had the highest field overlap. The sensor structure can be easily fabricated on a chip, and it shows a sensitivity of 1670 nm/RIU [59]. A wearable LSPR strain sensor based on graphene oxide and AuNPs was demonstrated for the monitoring of human mobility and is depicted in Figure 6b. This sensor has a high gauge factor of 52.5 and linearity over a wide strain range of up to 25.4% [60]. A wearable plasmonic metasurface with a SERS activity-based sensor was demonstrated for molecular fingerprint detection on bio-interfaces. This integrated sensing platform is fabricated by the integration of a flexible SERS-active plasmonic metasurface that serves as the key sensing component and a flexible electronic system capable of automatically extracting sweat and analytes from the body.

Various properties of the skin patch, including pinch, twist, waterproof, stretching, and compressing properties, have been studied in detail. A structure is fabricated using a 6 µm copper film on a silica wafer with a 10 µm cured PDMS layer as a temporary adhesive layer. The patch shows the different properties in different positions on the skin. This integrated sensor has a detection limit of 0.01 nM [61]. A plasmonic paper-based microfluidics sensor using label-free SERS was demonstrated to detect uric acid in the sweat sample at concentrations of 20 to 100 µM.

Raman spectroscopy is chosen to avoid the difficulties associated (degradation with time) with currently used bio-receptors which include antibodies, enzymes for specificity. The structure involves a few functional layers: double-sided adhesive with an inlet, a laser blocker, a paper microfluidic channel, Au-nanorod-based plasmonic paper, and a polydimethylsiloxane encapsulation layer at the top. The concentrations of uric acid can be calculated using the SERS intensity ratio [62,63].

#### 3.2.4. Photoplethysmography Based Sensors

**Figure 7:**
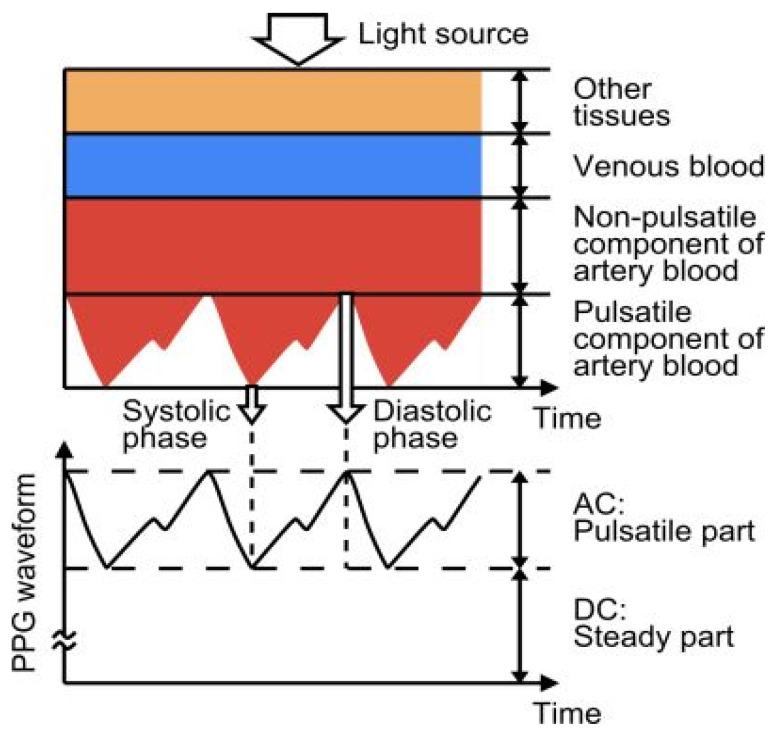
Photoplethysmography technique principle, structure, and output. Reprinted with permission from Electron, Copyright 2014, MDPI [65].

Photoplethysmography (PPG) detects variations in the volume of blood flow as detected by the light intensity spectrum. As light passes through the various biological components of tissue, including skin, bone, and arterial and venous blood, these components can absorb light, thereby altering the blood flow in the arteries and arterioles [64]. The output of a photoplethysmographic sensor consists of direct and alternating current, with direct current indicating the transmitted or reflected optical signal from the tissue, which is dependent on the tissue’s structure and the average arterial and venous blood volume. Alternating current represents changes in blood volume between the systolic and diastolic phases of the cardiac cycle, and its fundamental frequency is dependent on heart rate. Photoplethysmography is a popular method for monitoring heart rate due to its user-friendliness, low cost, and ease of operation. A standard PPG device includes both a light source and a photodetector. Thomas devised a method for measuring the electrocardiogram and photoplethysmogram using these two signals, pulse transit time through which systolic and diastolic as depicted in Figure 7. In this method, a nine-axis MEMS inertial sensor along with green LEDs was added to the photoplethysmography device to sense body measurements and detect posture. A single green LED, switched by a microcontroller-based sensor, was developed to mitigate motion artifacts by applying two reflective pulse signals [65].

#### 3.2.5. Interferometric Sensors

A single-mode hetero-core fiber-optic sensor-based textile was reported to monitor the heartbeat and respiration based on chest movement. The structure of the sensor consists of a single-mode fiber with a core and clad diameters of 9 and 125 μm and a short segment of fiber with diameters of 5 and 125 μm (hetero-core). The proportion of transmitted light that leaks into the cladding varies with the soft bending of the hetero-core fiber. The optical fiber of 1.3 mm diameter was woven with wool yarn in a twisted manner. Heartbeat and respiration were measured using a light-emitting diode with a wavelength of 1.31 μm, a photodiode, and an analog-to-digital converter [66].

**Figure 8:**
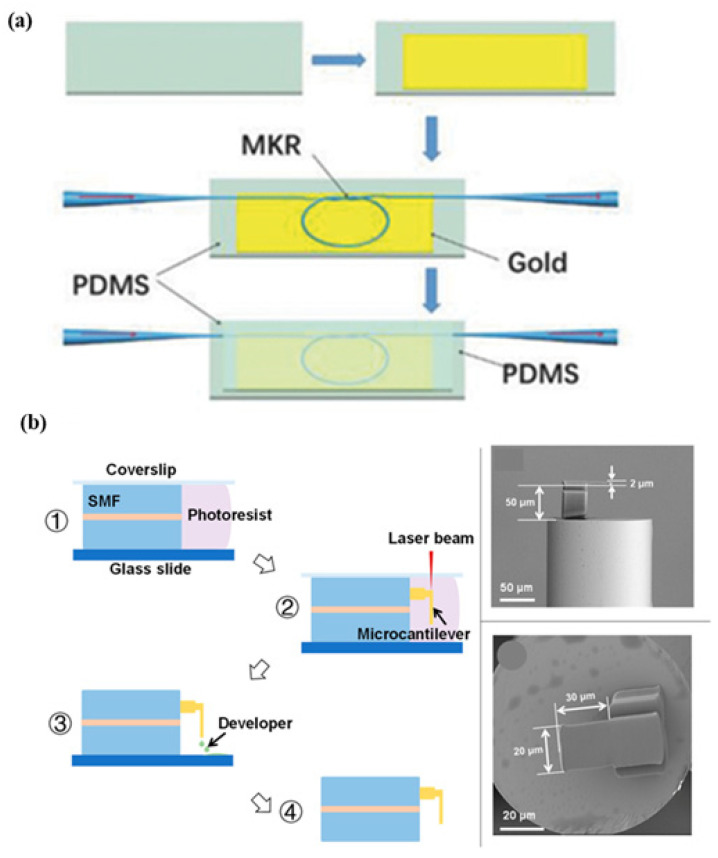
Schematic of various fiber-based wearable sensors. (**a**) Hetero-core optical fiber for respiration and heartbeat measurement. Reprinted with permission from Advanced Materials Technologies, Copyright 2018, Wiley [67]; (**b**) a respiration sensor using Fabry–Perot interferometry, fabrication steps of fiber-tip micro-cantilever that involve femtosecond laser and scanning electron microscopy of the fabricated wearable sensor. Reprinted with permission from Biosensors, Copyright 2018, MDPI [68].

A hybrid plasmonic microfiber knot resonator fixed on Au-film implanted in a 500 µm-thick polydimethylsiloxane that demodulates the sensing signals was demonstrated to detect the finger pulse, as shown in Figure 8a. The MZI polarization controller is used to adjust the polarization state in order to obtain stable and clear interference fringes [67]. A Fabry–Perot interferometry-based fiber sensor is fabricated for respiration rate and its different pattern measurement with a sensitivity of 0.8 nm/(m/s). The structure consists of a microcantilever fabricated on the tip of a fiber using two-photon polymerization microfabrication, as shown in Figure 8b. The working principle involves the reduction in the cavity length of the Fabry–Perot micro-interferometer to monitor blood pressure [68]. Zhu et al. fabricated a wavy microfiber-based wearable sensor using a bottom-up approach. To fabricate the sensor, tapered single-mode fiber is encapsulated in a few micrometers of thick polydimethylsiloxane film to fabricate the sensor. A sensitivity of 257 per unit strain was achieved using a microfiber sensor [69]. A fiber Bragg grating (FBG) [70]-based sensor was developed for pulse measurement for cardiovascular disease diagnosis using the concept of lever amplification that provides a sensitivity of 8.236 nm/N. The pulse waveforms at different positions and depths of the radial artery in the wrist were measured [71]. An MZI wearable fiber sensor was demonstrated for respiration rate and tidal volume. Analysis was performed using two core modes, LP01 and LP11, with a sensitivity of 8.53 dB/m^−1^. To fabricate the structure, a few-mode fiber with an ‘L’ length is inserted between two SMF-28e single-mode fibers [72]. A fiber-optic-based Fabry–Perot interferometer was reported to measure the heart rate and blood pressure [73]. Presti et al. demonstrated an FBG-based wearable sensor for monitoring respiration and cardiac actions. The structure of the sensor consists of a grating encased in silicone rubber using a dragon skin, with a sensitivity of 0.125 nm·mε^−1^. The sensor can be used to measure temperature, relative humidity, and water immersion in harsh environments [74,75]. It was demonstrated that a Fabry–Perot sensor based on ethyl alpha-cyanoacrylate can monitor the heart rate in various positions on the wrist, chest, and neck. The structure is composed of a capillary tube with two fiber cantilevers and ethyl alpha-cyanoacrylate as a binder and bracket. Due to its low Young’s modulus, ethyl alpha-cyanoacrylate is utilized to detect lower vibration frequencies with high sensitivity. The strain sensitivity of 2.57 pm/µN for the frequencies lying in the range of 1–3 Hz [76]. A double-core photonic crystal fiber-based sensor with interrogation based on low coherence interferometry was reported for temperature measurement [77,78].

#### 3.2.6. SERS Sensors

**Figure 9:**
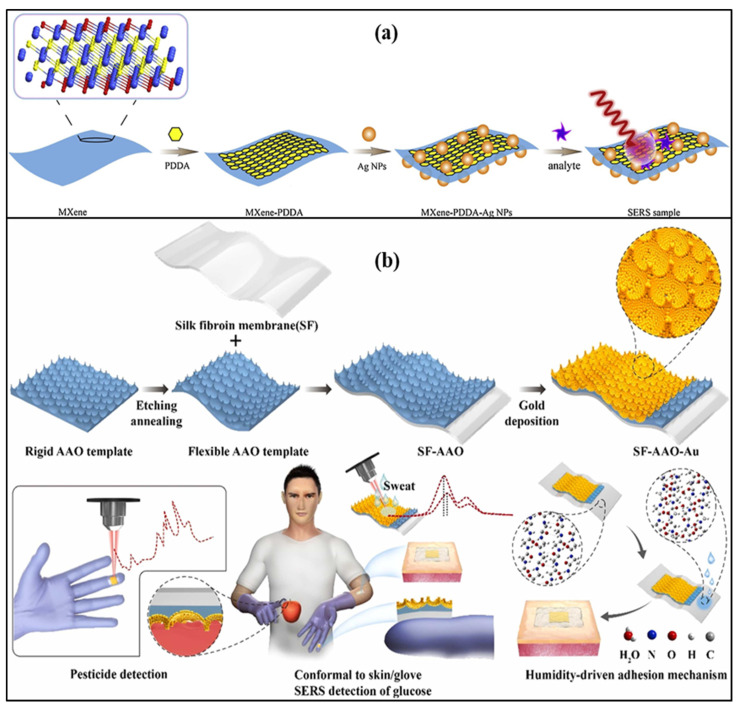
Schematic of (**a**) Ti_3_C_2_T_x_ Mxenes and AgNPs-based SERS label-free sensor. Reprinted with permission from Sensors and Actuators B: Chemical, Copyright 2021, Elsevier [80]; (**b**) smooth silk fibroin film and large-area gold nano-islands-based SERS sensor. Reprinted with permission from Sensors and Actuators B: Chemical, Copyright 2021, Elsevier [81].

An Ag nanowire, silk fibroin protein film-based label-free SERS patch type was demonstrated to monitor the drug molecule in the sweat sample. Nanometer-thick silk fibroin film absorbs molecules from the skin of the human body. Ag nanowire enhances the SERS signal and creates a transparent dermal protecting layer that allows laser penetration through Ag nanowire. 2-fluoro-methamphetamine was used to check the feasibility of the developed SERS sensor [32]. An omnidirectional plasmonic nano-voids array-based SERS sensor is demonstrated to detect the dopamine in the sweat sample by mounting it on the forehead of a person. The sensor provides an ultralow detection limit of 10^−16^ M for the sweat concentrations. This sensor was also used for hydrogen sulfide gas sensing by fixing a patch on a mask with a limit of detection of 1 ppb [33]. A flexible, transparent, and smooth silk fibroin film and large-area gold nano-islands-based SERS sensor was reported for the detection of 4-aminobenenethiol. The nano-islands offer a dense nano-gap structure and excellent spatial uniformity, resulting in sensitive detection. SERS intensity of nano-island was found to be 14 times that of the conventionally used smooth silicon surface, which also improves sensitivity and detection limit [79]. Liu et al. reported a Ti_3_C_2_Tx Mxenes and AgNPs-based SERS label-free sensor based on an electrostatic self-assembly method to detect dopamine concentrations of 5–500 µM, as shown in Figure 9a.

The cationic polymer poly (diallyl dimethyl ammonium chloride) was used to change the nature of the MXene surface, as functional groups in MXene and AgNPs coated with citrate are negatively charged in nature. It provides a detection limit of 100 µM [80]. A SERS-based wearable sensor that consists of Au nanoparticles, silk fibroin, and aluminum oxide that can be attached to gloves with the help of glue was demonstrated for sweat glucose detection. In the sensor structure, silk fibroin, an aluminum oxide cavity, and AuNPs are used, which act as a flexible support template and help in the generation of plasmons, as shown in Figure 9b. It can measure the glucose concentration in the range of 100–10,000 μM with a detection limit of 100 μM. This lab-on-glove sensor was also used to explore the Thiram bactericide, which causes several diseases related to the skin, liver, and eye [81].

#### 3.2.7. Other Optical Sensor Structures

A PDMS film-based stretchable skin-like wearable optical sensor using silica micro-/nanofibers that provide flexibility for stretching or bending was demonstrated to monitor the temperature in the range of −20 to 130 °C, respiration, and arm motion. PDMS has a large thermo-optic coefficient that provides high sensitivity for temperature sensing. This sensor is able to provide a gauge factor as high as 675 in the case of strain sensing, sensitivity in the range of 0.01–0.47 per degree for bend sensing, and a high resolution of 0.013 °C for temperature detection [82]. Elsherif et al. developed a phenyl boronic acid-functionalized hydrogel specific for a glucose-based photonic sensor structure for diabetes. Phenyl-boronic acid provides stability and affinity towards diol for glucose monitoring in the continuous mode. The periodicity of the photonic structure was 1.6 μm for the glucose concentration, which lies in the range of 0 to 50 mM and provides a sensitivity of 12 nm m/M [83]. Using the strain property, an FBG-based skin sensor with a stretchable polydimethylsiloxane substrate was created to monitor human activity, such as respiration, phonation, facial expression, and joint movement. Using a single-walled carbon nanotube mode-locked fiber laser, the Bragg wavelength shift was measured [84]. Table 1 summarizes the various wearable optical sensors based on different mechanisms, their limits of detection, and their range values. A nanowire-based, water-soluble polyurethane-MXene strain sensor was demonstrated for monitoring crack propagation. Six fiber-strain sensors are woven onto different regions of a corset’s back to develop a sensor that is constructed using smart clothing. The sensors measure the strains due to different body movements, which include neck motion, leg motion, right shoulders, arm movement, and back bending. The sensor is able to provide the sensitivities measured in terms of gauge factors >100 with a response time of 344 ms [66]. A wearable graphene-coated fiber sensor to monitor human motion during basketball and soccer games was created. A double-covered yarn is used as the elastic scaffold, and the yarn is comprised of polyurethane core fiber and polyester fibers winding around the polyurethane core in a helical form [85]. Xiang et al. developed a fiber Bragg grating-based wearable sensor for temperature monitoring fixed onto textiles. This structure can detect temperatures in the range of 20−100 °C [86].

### 3.3. Samples: BioFluids

Different body fluids that include tears, blood, ISF, urine, and saliva are important biomarkers that indicate the physiological status and diagnosis of various diseases [89]. Urine and saliva are more accessible but have limited biomarkers and variable concentrations. Sweat is a biofluid generated by three types of sweat glands present in the human body, which include eccrine, apocrine, and apoeccrine, and contains numerous important biomarkers that could be detected and assessed through non-invasive collection and biochemical analysis. Out of the three glands, eccrine produces the majority of the sweat produced by the body and is present everywhere on the body [90,91,92,93,94,95]. All the details of fluid constituents in terms of metabolites, electrolytes, and pH values are summarized in Table 2. Glucose, lactate acid, and cortisol in sweat indicate different diseases such as diabetes, fibrosis, stress ischemia and hyperglycemia, obesity, and bilateral adrenal hyperplasia. The presence of different components indicates the following diseases and disorders: Sodium ions (Na^+^): hypernatremia, increased heart rate, potassium ions (K^+^): renal failure, hyperkalemia, hypokalemia, copper (Cu^2+^): disorders of the bones, anemia, edema, toxicity, zinc (Zn^2+^): poisoning, mercury (Hg^2+^): renal failure, gastrointestinal hemorrhage, chronic nephritis, and uremia, (cadmium) Cd^2+^: lung cancer, hypertension, bronchitis, emphysema, and poison, (lead) Pb^2+^, calcium ions (Ca^2+^): rhabdomyolysis, primary hyperparathyroidism, immunocompromised, hypocalcemia, muscle spasm, and ammonium (NH^4+^) ions: liver disease, adrenal hyperplasia, ascorbic acid: tumors, stones, heart diseases, carcinoma, kidney disease, thrombosis, caffeine: panic attack, heart disease, insomnia, ethanol, uric acid: metabolic myopathies, gout, kidney disease, pH: fungal infections, hydration, skin-related diseases, protein: cardiovascular disease, melanoma [96]. ISF is generated in the dermis through blood transcapillary filtration and can be used as a blood substitute for medical health status indicators; its collection is easy and non-coagulating in nature. Saliva consists of fluoride, Na^+^, urea, K^+^, uric acid, cholesterol, cadaverine, Cl^−^, putrescine, Ca^2+^, Mg^2+^, creatinine, ${\mathrm{HPO}}_{3}^{2-}$, fatty acids, and protein, ${\mathrm{HCO}}_{3}^{-}$. It is also used to monitor bacteria and metabolites. Contact lenses have attracted considerable interest as a platform for in situ biosensing of tear fluid with a pH of 6.5 to 7.6 that contains glucose, lactate, proteins, ascorbate, Na+, Cl^−^, and K^+^ [97,98,99]. Urine can be collected non-invasively, and the amount of fluid is significant and plays an important role in monitoring health and various diseases. As it is difficult to calibrate the urine concentrations, which are limited by an individual’s hydration level, urine-based wearable sensors suffer difficulties in practical applications. Blood-based wearable sensors have not been commercialized on a large scale due to their invasive and skin-piercing nature for sample collection [100]. Piezoelectric materials have been widely explored for self-powered sensors and energy-harvesting solutions in bio-integrated devices because of their excellent capability to generate an electric charge in response to mechanical deformations. Table 3 compares the various advantages and disadvantages associated with optical and conventional wearable sensors to monitor health.

## 4. Future Prospective

Each type of sensor has numerous advantages and disadvantages. Different biofluid-based sensors suffer from several difficulties that include low sweat rates, sensor stability in harsh environments, skin contamination, fresh sweat collection, sample evaporation, low-concentration molecular detection, and calibration rendering. Using wearable technologies, it has recently been possible to detect nucleic acids, such as SARS-CoV-2 guide RNA in a breath sample via freeze-dried reactions in a face mask, and CRISPR-Cas12a-specific high-sensitivity enzymatic reporter unlocking sensing. Tears contain a large array of protein biomarkers, such as antibodies, neuropeptides, and protective proteins, which can be utilized to diagnose ocular disorders such as dry eye syndrome, diabetes, systemic sclerosis, cystic fibrosis, cancer, and Parkinson’s disease [105]. Optical sensors provide the advantages of easy fabrication methods, simple skin integration, and no requirement for extra electronic components within the body [106]. Among the current wearable sensing techniques, enzymatic processes, ionophoreion interactions, and direct molecular oxidation/reduction have been successfully researched and can monitor a limited number of targets directly. Monitoring fluids, such as circulating metabolites, blood cells, minerals, hormones, and proteins, plays an important role in the early diagnosis of diseases that cannot be detected with current wearable sensing techniques. Molecular recognition based on bio-affinity receptors and molecular switches is an emerging wearable biosensing technique that holds great promise for the detection of a broad spectrum of analytes in biofluids. However, there are numerous challenges relating to stability and reversibility that require further study. The strength of the measured PPG signal is highly dependent on location, limiting the precision and repeatability of the wearable PPG device when customers do not have the sensing devices mounted securely or consistently, which is one of the primary sources of disagreement. Wearable sensors necessitate appropriate power sources in order to operate at their maximum potential. Emerging sensors based on energy harvesters that can convert one form of energy to another are being extensively researched for numerous uses. The combination of biosensors with functional micro/nanomaterials enables real-time biophysical and biochemical monitoring of health status [107,108]. A real-time measurement of the moisture levels of human skin can be used to monitor respiration and water evaporation, which is crucial for disease state monitoring. [109]. Despite several breakthroughs, the development of wearable sensors for health monitoring faces numerous obstacles. These obstacles are associated with various aspects, including material advancements, fabrication techniques, precision, and data processing. Various materials have been used in wearable health sensors so far, but additional efforts are required to develop stretchable semiconductors and dielectrics. Due to the minute fluctuations in human body temperature, wearable sensors must possess not only high precision, high sensitivity, and high resolution but also mechanical flexibility and biocompatibility. Given the constraints of microfabrication techniques and tools for biodegradable materials, this can be regarded as a hard topic. Soft materials have a limit on the depth of structures that can be etched at a given rate. Therefore, thick-film device fabrication is problematic. In addition, biopolymeric materials cannot be processed at high temperatures, as this can cause complications during fabrication.

The challenges associated with wearable optical sensors include selection, the development of suitable and comfortable materials, fabrication processes, and accuracy. More stretchable materials that include semiconductors and dielectrics are required to explore. Multi-modality sensing is required, which includes the use of a single structure to detect multiple stimuli and fix multiple sensors on a single platform. Hormone sensing should be focused on in the case of wearable sensors due to the reduced reliability of non-invasive biofluids as it involves preconcentration techniques and dilution of the analytes. In the case of fiber-optic-based sensor designs such as Bragg grating, Mach–Zehnder, and Fabry–Perot interferometers, light sources are required, and mass data due to high-frequency physiological signals and modulation devices make the sensor bulky in size and expensive. Wearable sensors have various applications in aerospace, including eye-tracking glasses, radio headphones, oxygen masks, and helmets for airplane manufacturing applications, cardiac monitoring devices, in-flight monitoring, and air quality management [17,110,111,112].

## 5. Conclusions

In this review, various optical-based wearable sensors have been discussed in detail. Optical sensors for wearables are recently explored techniques due to developments made in the fields of fabrication, synthesis, and biosensors. Wearable sensors have been explored for heart rate, pulse rate, ECG, blood pressure, and body temperature for health monitoring purposes for personalized medicine and cancer biomarkers in the case of different diseases and can provide a promising solution to identify various infected patients in cases of diseases such as COVID-19 infection Among electrochemical and optical sensors, electrochemical sensors are commonly used due to their ease of fabrication and commercialization. In the case of optical sensors, high sensitivity, high long-term stability, and high-frequency data collection are difficult to achieve with a miniaturized external wearable system. Colorimetric, SERS, fluorescence, plasmonics, and interference-based wearable sensors are in the development stage. Out of all SERS, including photoplethysmography, colorimetry, fluorescence, plasmonics, and interference sensing strategies, photoplethysmography shows better performance and is widely commercialized for various applications. The intensity of the photoplethysmography signal varies according to the position and shows large variations in the results, which limit its sensitivity, accuracy, and repeatability. Future research should investigate the viability of affinity-based wearable sensors for a variety of disease and health monitoring variables. Batteries or near-field communication are used to provide power to existing wearable sensors, whose long-term, continuous usability is limited. Self-powered optical wearables, harvesting energy from body fluids, human motion, or sunlight, are attractive candidates for next-generation wearable biosensors.

## Figures and Tables

**Table 1:** Summary of various commercially available wearable optical sensors.

Mechanism	Nanomaterials	Analyte	Linear Range	LoD	Ref.
**SERS**	Ag and MoS_2_	Rhodamine 6G	1000–10,000 μM	10^−13^ M	[35]
**Colorimetric**	AuNPs	Chloride	0–125 mM	n.r. ^a^	[47]
**Colorimetric (sweat)**	AuNPs	Chloride	25–100 mM	0.06 mM	[48]
		Glucose	25–100 mM		
		Lactate	5–20 mM		
		pH	5–6.5		
		Temperature	25–37 °C		
**Colorimetric**	n.r. ^a^	Glucose	0–2.0 μM	27 μM	[49]
		Nitrite	0–400 μM	7 μM	
**Colorimetric**	AuNPs	Glucose	0.01–0.15 mM	0.01 mM	[51]
**Colorimetric and Fluorescence**	n.r. ^a^	SARS-CoV-2	n.r. ^a^	2.7 fM	[52]
**Fluorescence**	n.r. ^a^	Chloride	0 to 200 mM	0.1 mM	[55]
**Fluorescence**	n.r. ^a^	Glucose	10–250 μM	7 μM	[56]
		Lactate	1.0–12.5 mM	0.4 mM	
		Chloride	10–100 mM	5 mM	
**Fluorescence**	n.r. ^a^	Glucose	23 μM–1.0 mM	9.3 μM	[58]
**LSPR**	AuNPs	A549 cells	n.r. ^a^	n.r. ^a^	[59]
**SERS**	Ag nanocube	Nicotine	10–100 nM	0.01 nM	[61]
**SERS**	Au nanoislands	Para-aminothiophenol	200 nM–10 µM	11.4 µM	[79]
**SERS**	Ti_3_C_2_T_x_ MXenes	4-mercaptobenzoic acid	5–500 μM	1000 µM	[80]
**SERS**	AuNPs	Glucose	100–10,000 μM	16.8 μM	[81]
**Photonic microstructure**	n.r. ^a^	Glucose	0−50 mM	n.r. ^a^	[83]
**Colorimetric**	AuNPs	Lactate	10−30 mM	0.06 mM	[87]
**SERS**	Ag nano-mushroom	Urea, lactate, pH	1–10 mM	n.r. ^a^	[88]
			1–10 mM		
			5.5–7.0		

^a^ Not reported.

**Table 2:** Details of constituents and pH values of different biofluids.

Fluid Type (pH Values)	Constituents	Ref.
Sweat (3.0–8.0)	Glucose (10–200 μM), lactate acid (5–20 mM), Na^+^ (10–100 mM), Cl^−^ (10–100 mM) K^+^ (1–18.5 mM), Cu^2+^ (100–1000 μg/L), Zn^2+^ (100–1560 μg/L), Hg^2+^ (<100 μg/L) Cd^2+^ (<100 μg/L), Pb^2+^ (<100 μg/L), Ca^2+^ (0.41–12.4 mM) and NH^4+^ (0.1–1 mM) ions, cortisol (8–140 ng/Ml), adrenal hyperplasia, ascorbic acid (10–50 μM), caffeine, ethanol (2.5–22.5 mM), uric acid (2–10 mM), pH (3.0–8.0), proteins	[6,90,91,92,93,94,95]
Tears (6.5–7.6)	Glucose (0.1–0.6 mM), lactate (1–5 mM), urea (6 mM), proteins (5–11 mg/mL), ascorbate (11–23 µM), Na^+^ (120–165 mM) Cl^−^ (118–135 mM), K^+^ (15–42 mM) Mg^2+^ (0.5–1.1 mM)	[96,97,98]
Interstitial fluid (7.35–7.45)	Mg^2+^ (0.887 mmol/L), and Ca^2+^ (2.365 mmol/L), proteins (20.6 g/L)	[99]
Saliva (6.0–7.0)	Fluoride (0.015–0.045 mg/100 mL), Na^+^ (0–20 mg/100 mL), K^+^ (60–100 mg/100 mL), urea (0.12–2.0 µg/mL), cholesterol (0.14 µg/mL), Cl^−^ (50 mg/100 mL), Ca^2+^ (2.2–11.3 mg/100 mL), phosphate (6.1–71 mg/100 mL), creatinine (0.05–0.2 mg/100 mL *^a^), mucin (1.92 µg mL^−1^), ${\mathrm{HPO}}_{3}^{2-}$, total protein (0.9 mg mL^−1^), ${\mathrm{HCO}}_{3}^{-}$ (1–60 mM), cortisol (0.1–0.2 µg/mL)	[7,49,100,101,102]
Urine (5.5–7.0)	Creatinine (601–2936 mg/d), uric acid (16–750 mg/d), Cl^−^ (40–224 mmol/d), Na^+^ (41–227 mmol/d), K^+^ (17–77 mmol/d), Mg^2+^ (51–269 mg/d), sulphate (7–47 mmol/d), ammonium (15–56 mmol/d), phosphate (20–50 mmol/d), urea (10–35 g/d)	[7,30,103,104]

* Conversion (^a^ 1 mg/100 mL = 10 µg/mL).

**Table 3:** Comparison of optical and conventional wearable sensors for different health monitoring applications.

Types of Sensors	Advantages	Disadvantages
Optical sensors	Low-cost fabrication, naked-eye estimation Easy design, quick optimization, and versatile Electromagnetic interference immune Miniaturization, detection of nano-volumes and integration with fiber platform High sensitivity Usable in harsh environments and corrosion resistant	Dye biocompatibility in fluorescence and colorimetric sensors Requires bulky UV lamps or fluorescence spectrophotometers Requires specially designed probes Need for special algorithm development for smartphone-based approach Bulky size
Electrochemical Sensors	Low waste fabrication Possibility of large-scale production	Requires external power sources Degradation of electrode and other constituents with passage of time Requires amplification techniques for highly sensitive signals
Piezoelectric sensors	Simple fabrication and high frequency response and Broad sensing range	Charge leakage and only possibility of dynamic sensitivity

## Data Availability

Not applicable.
